# Epidemiology and lagged climatic drivers of pediatric viral lower respiratory tract infections in Tianjin, China (2023–2025)

**DOI:** 10.3389/fcimb.2026.1893926

**Published:** 2026-09-24

**Authors:** Shiyin Mu, Jia Zhai, Fei Gao, Yongsheng Guo, Jiao Li, Run Guo, Xingda Wen, Yingxue Zou

**Affiliations:** 1 Department of Respiratory of Ma-Chang, Children’s Hospital, Tianjin University/Tianjin Children’s Hospital, Tianjin, China; 2 Children’s Hospital, Tianjin University, Tianjin, China

**Keywords:** lagged climate analysis, meteorological factors, pediatric lower respiratory tract infection, respiratory virus surveillance, Tianjin, time-series regression

## Abstract

**Background:**

Following the lifting of pandemic-era restrictions, China experienced a sharp resurgence in pediatric respiratory infections; however, post-pandemic epidemiological and climatic data on lower respiratory tract infections (LRTIs) from the temperate continental climate of northern China remain scarce.

**Methods:**

In this retrospective, single-center surveillance study, we characterized the post-pandemic epidemiology of six respiratory viruses in children hospitalized with LRTI in Tianjin and assessed their pathogen-specific, lagged climatic associations. Children aged 0–18 years hospitalized between January 2023 and December 2025 were tested by targeted next-generation sequencing for human rhinovirus (HRV), adenovirus (ADV), respiratory syncytial virus (RSV), human bocavirus (HBoV), influenza virus (IFV), and human metapneumovirus (HMPV). Pathogen–climate associations were estimated using a quasi-Poisson model that adjusted for seasonality, secular trend, and lagged meteorological exposures.

**Results:**

Among 2,597 virus-positive cases, the leading pathogens were HRV (30.7%), ADV (26.6%), and RSV (20.6%), followed by HBoV (12.1%), IFV (6.0%), and HMPV (4.0%). Age distributions differed by pathogen (p<0.05): RSV and HBoV predominated in infants and toddlers, HRV and HMPV in preschoolers, ADV in school-aged children, and IFV across the older preschool-to-school-age range; males predominated for all viruses except IFV. Five viruses peaked in December, whereas HBoV peaked uniquely in July. After adjustment for seasonality and secular trend, the unadjusted associations of IFV with humidity and temperature disappeared, indicating that they largely reflected shared seasonality. In contrast, RSV retained associations with temperature (RR 3.87, 95% CI 1.39–10.76) and wind speed (RR 5.46, 95% CI 2.39–12.47) at a 1-month lag, and HMPV with temperature (RR 13.3, 95% CI 1.64–108.3; imprecise given its low case count); HRV, ADV, and HBoV showed no independent climatic associations. Co-infections occurred in 116 cases (4.5%), most frequently HBoV+HRV (23.3%).

**Conclusion:**

Post-pandemic pediatric viral LRTI in Tianjin shows distinctive pathogen-specific profiles in age, seasonality, and climatic sensitivity. The summer peak of HBoV and the seasonally independent climatic drivers of RSV and HMPV provide a preliminary basis that, if confirmed with higher-resolution data, could inform pathogen-tailored, climate-informed surveillance in northern Chinese megacities.

## 1 Introduction

Lower respiratory tract infections (LRTIs)—comprising pneumonia, bronchiolitis, and related lower-airway syndromes—remain leading causes of pediatric hospitalization and mortality in children under five worldwide, with viruses accounting for the overwhelming majority of cases (Nair et al., 2010; Mulholland, 2003; Li et al., 2022). In contrast to generally self-limiting upper respiratory infections, viral LRTIs account disproportionately for admissions requiring supplemental oxygen and intensive care; moreover, certain pathogens, notably RSV and HRV, are implicated in long-term sequelae including recurrent wheeze and asthma (Li et al., 2022; Bizot et al., 2021; Royston and Tapparel, 2016). In China, the burden is amplified by dense urban populations, overlapping pathogen circulation, and the high contact rates among school-aged children, all of which sustain substantial LRTI-related hospitalizations (Cui et al., 2024; Liu et al., 2025). Following the lifting of pandemic-era non-pharmaceutical interventions, several Chinese regions reported atypical, out-of-season resurgences of RSV, Mycoplasma pneumoniae, and ADV (Del Riccio et al., 2024; Liu et al., 2025). These have been attributed both to an immunity gap accumulated during 2020–2022 and to the re-establishment of pre-pandemic transmission networks (Cohen et al., 2021; Li et al., 2024). Despite this evolving landscape, most national surveillance efforts remain narrowly focused on classical pathogens (RSV, ADV, IFV) or aggregate all respiratory infections irrespective of anatomical site. Consequently, the post-pandemic, multi-pathogen epidemiology of viral LRTI specifically in children—particularly at the city level, where targeted surveillance is most actionable—remains inadequately characterized (Terliesner et al., 2022; Xia et al., 2026).

Ambient weather conditions are well-established modulators of respiratory virus transmission, operating through both physical and host-defense mechanisms (Baker et al., 2019; Xu et al., 2021). Cold, dry air favors the persistence and airborne dispersal of enveloped viruses such as RSV and influenza by stabilizing the lipid envelope, slowing droplet evaporation, and impairing upper-airway mucociliary clearance (Kudo et al., 2019; Lowen et al., 2007; Lowen and Steel, 2014). Non-enveloped viruses, including ADV and HBoV, whose protein capsids tolerate a wider range of environmental stressors, tend to circulate more evenly throughout the year (Chen et al., 2014b; Lion, 2014). Importantly, climate–virus relationships are non-linear and time-delayed; exposures occurring days to weeks before case surges often exert the dominant effect, a structure that makes distributed lag non-linear models (DLNMs) particularly suitable for fine-grained inference (Bhaskaran et al., 2013; Gasparrini et al., 2010; Gasparrini, 2011). A recent post-pandemic study from Wuhan, in central China, applied daily-resolution DLNMs to seven respiratory pathogens and identified distinct lagged climate patterns—most notably, humidity drops as 1–2-week antecedents of RSV surges and wind speed as a leading correlate of influenza activity (Li et al., 2025). Because only monthly-resolution meteorological data were available for the present 2023–2025 window (Section 2.3), a full DLNM cross-basis was not identifiable with the available degrees of freedom; we therefore adapted the same distributed-lag logic into a screening framework of single-lag quasi-Poisson models spanning 0–6-month lags, cross-validated against cross-correlation functions and, in a second step, adjusted for seasonality and secular trend within a unified model (Section 2.4). This approach is best viewed as a monthly-resolution approximation to the DLNM framework rather than a full non-linear cross-basis estimation.

However, few studies have systematically examined this multi-pathogen landscape in northern China, where the continental monsoon climate—characterized by cold, dry winters; hot, humid summers; and pronounced seasonal swings in temperature, humidity, and wind—differs markedly from the subtropical conditions of Wuhan and other southern cities (Chen et al., 2014a; Cui et al., 2015; Yin et al., 2023). Tianjin, a megacity of 13 million on the North China Plain, offers a particularly valuable setting to address this gap. Its local viral spectrum extends beyond the four classical agents to include three pathogens that are less consistently surveilled but contribute substantially to pediatric LRTI: HRV, HBoV, and HMPV (Feng et al., 2024; Jiang et al., 2016; Wang et al., 2022; Zhu et al., 2020). Prior work in this area has concentrated largely on southern China or on the classical pathogens (RSV, ADV, IFV), and the lagged climatic effects of the less consistently surveilled HRV, HBoV and HMPV under a temperate continental climate have not been systematically evaluated. Precisely how these three viruses, alongside the classical agents, track climatic exposures across different lag intervals in northern China therefore remains poorly characterized—a knowledge gap that has direct implications for the design of climate-informed early-warning systems in the region.

To address this, we analyzed three years of post-pandemic surveillance data (January 2023 to December 2025) from a tertiary children’s hospital in Tianjin, encompassing 2,597 virologically confirmed pediatric LRTI cases caused by six respiratory viruses. Our objectives were: (i) to describe the demographic, temporal, and specimen-type distributions of each pathogen; (ii) to characterize co-infection patterns; (iii) to test pathogen-specific associations with monthly meteorological variables using Spearman correlation and season-stratified analyses; and (iv) to evaluate lagged climate effects across 0- to 6-month intervals using cross-correlation functions and a unified quasi-Poisson generalized linear model that simultaneously adjusts for seasonality and secular trends. Finally, we discuss the practical implications of our findings for climate-informed early-warning systems and pathogen-tailored preventive strategies in northern Chinese megacities.

## 2 Materials and methods

### 2.1 Study design, setting and population

We conducted a retrospective single-center surveillance study at the Department of Respiratory Medicine, Tianjin Children’s Hospital (Ma-Chang campus), the only tertiary-A comprehensive children’s hospital in Tianjin. The hospital is the principal pediatric referral center for Tianjin municipality and, for selected specialties, draws patients from across the wider North China Plain; publicly reported hospital-wide figures indicate roughly 17,000–20,000 pediatric admissions per year across all departments. Our cohort of 2,597 virologically confirmed viral LRTI cases represents the subset of hospitalized LRTI cases that were both tested and found positive for one of the six target viruses, not the total burden of pediatric LRTI hospitalization in Tianjin. A precise, denominator-based estimate of the proportion of all Tianjin pediatric LRTI hospitalizations captured by this cohort is not available, because no citywide pediatric LRTI hospitalization registry exists for comparison. As a tertiary referral center, the cohort is likely enriched for more severe disease and for cases referred from primary and secondary facilities; this referral pattern, and the resulting limit on generalizability to primary care, is revisited in the Discussion (Section 4.6).

Eligible participants were children aged 0–18 years hospitalized with a clinical diagnosis of LRTI between 1 January 2023 and 31 December 2025, from whom a respiratory specimen was submitted for routine viral testing. LRTI was defined clinically, following the 2019 Chinese Pediatric Society guideline for community-acquired pneumonia (Subspecialty Group of Respiratory Diseases, 2019), as cough or fever (axillary temperature ≥38 °C) together with at least one of: tachypnoea, wheezing, crackles on auscultation, or imaging evidence of pneumonia or bronchiolitis. This definition captures the clinical syndromes of pneumonia and bronchiolitis and excludes isolated upper respiratory tract infection. We excluded children with pre-existing chronic respiratory disease, congenital airway malformations, documented immunodeficiency, active malignancy, or concurrent infection by pathogens outside the predefined six-virus panel. This last criterion operated at the level of the child rather than of the laboratory result: whenever the tNGS report carried a Class A detection of a bacterial, atypical or non-panel viral pathogen alongside one of the six target viruses, the case was removed from the cohort at eligibility screening, so no such child contributes to any analysis reported here. For viruses prone to subclinical carriage, chiefly HRV and HBoV, a detection was considered clinically causative only when the tNGS reporting algorithm assigned a pathogenicity Class A grade, the clinical picture matched the corresponding viral syndrome, and other plausible infectious causes had been clinically excluded (residual misclassification risk for these two viruses is discussed in Section 4.6). For descriptive analyses, children were stratified *a priori* into four age categories: infants (<1 year), toddlers (1–2 years), preschoolers (3–5 years) and school-aged children (≥6 years). All records were de-identified before analysis. The study followed the Declaration of Helsinki and was approved by the Institutional Ethics Committee of Tianjin Children’s Hospital (approval number 2024-LXKY-023); written informed consent was waived given the retrospective design and full de-identification of the dataset.

### 2.2 Pathogen detection

Respiratory specimens (sputum, the predominant type; bronchoalveolar lavage fluid, BALF; nasopharyngeal swabs; and oropharyngeal swabs) were collected following standardized clinical protocols, placed into viral transport medium, and transported within two hours at 2–8 °C to the clinical laboratory. Total nucleic acids were extracted on an automated platform following the manufacturer’s instructions. All specimens were tested with the same commercial targeted next-generation sequencing (tNGS) panel, which simultaneously screens for respiratory bacteria, viruses, atypical pathogens and antimicrobial-resistance markers. Library construction, ultra-multiplex amplification of pathogen-specific genomic regions and Illumina-compatible high-throughput sequencing were performed following the manufacturer’s recommended workflow, with reads aligned against a curated pathogen reference database (Li et al., 2025; Shang et al., 2025). The vendor’s reporting algorithm assigned each detected organism a clinical pathogenicity grade, from Class A (highest confidence) through lower-confidence grades. The panel was introduced during 2023 and reached full clinical implementation in the fourth quarter of that year, after which testing volume remained broadly stable through 2024 and 2025. Monthly counts of tNGS requests could not be retrieved from the laboratory information system for the whole study window, so this account of testing volume rests on the laboratory’s implementation record rather than on an auditable series of test counts, and it should be read as such.

The grade is a composite call rather than a single read-count cut-off. It is generated by an integrated scoring system that combines normalized read counts (reads per million, RPM), the breadth of genome coverage achieved across the targeted regions, and species-level annotation of pathogenicity against a curated reference database; Class A is assigned to organisms that meet the pre-specified RPM and coverage criteria for a clinically significant pathogen. The numerical RPM and coverage cut-offs behind the grade are proprietary to the assay vendor and are not released to the ordering laboratory; we are therefore unable to report them, and the grade as issued was the only form in which this information was available to us. Grading was prospective in the sense that it was performed by the clinical laboratory at the time the routine diagnostic report was issued and used for patient care, and the research team did not re-adjudicate the classifications retrospectively. No Class A detection was subsequently excluded on the grounds of clinical mismatch, because the Class A flag served as an inclusion criterion for the six-virus panel rather than as the sole determinant of clinical causality; for HRV and HBoV, the additional clinical adjudication described in Section 2.1 was applied instead.

The full panel detects a broader range of organisms than the six viruses analyzed here. Common bacterial pathogens reported by the full panel include Streptococcus pneumoniae, Haemophilus influenzae, Moraxella catarrhalis and *Staphylococcus aureus*; the atypical pathogens include Mycoplasma pneumoniae and Chlamydia pneumoniae; and respiratory viruses outside the six-virus panel, including parainfluenza viruses, seasonal coronaviruses and SARS-CoV-2, are likewise covered. Consistent with the eligibility criteria set out in Section 2.1, a child in whom any of these organisms was reported concurrently with one of the six target viruses was excluded from the cohort at screening rather than merely omitted from the analysis; the cases analysed here are therefore restricted to children whose Class A detections fell entirely within the six-virus panel. We restricted the present analysis to the six respiratory viruses (HRV, ADV, RSV, HBoV, IFV, covering both influenza A and B, and HMPV) flagged at the highest pathogenicity grade (Class A), for three reasons. First, these six viruses are the pathogens for which pathogen-specific climatic drivers are most biologically plausible and best characterized, allowing direct comparison with prior lagged-climate studies (Baker et al., 2019; Li et al., 2025; Xu et al., 2021). Second, restricting to Class A calls minimizes the inclusion of low-confidence or incidental detections. Third, bacterial and atypical pathogens are subject to different detection biases (for example, antibiotic pretreatment and colonization) and different climate–transmission mechanisms, and were therefore outside the scope of this analysis. Co-infections, defined as concurrent Class A detection of two or more of the six target viruses in the same specimen, were identified and are described in Section 2.4.

### 2.3 Meteorological data

Monthly city-wide meteorological summaries for Tianjin from January 2023 to December 2025 (n = 36 months) were obtained from the Tianjin Meteorological Bureau and comprised mean atmospheric pressure (hPa), mean wind speed (m/s), mean air temperature (°C), mean relative humidity (%), precipitation (mm) and mean daily sunshine duration (hours). Routine quality assurance included unit verification and column-order auditing across reporting periods. The meteorological series was time-aligned with the monthly aggregated viral case counts before analysis.

We used monthly rather than daily or weekly data because the records available from the Tianjin Meteorological Bureau for this study period were supplied at monthly resolution; daily or weekly city-wide summaries were not accessible for the full 2023–2025 window. Finer (for example, weekly) resolution would allow more precise estimation of short-lag effects and is standard in DLNM-based climate–infection studies (Gasparrini et al., 2010; Li et al., 2025); we discuss this resolution constraint further in Section 4.6.

### 2.4 Statistical analysis

Categorical data are presented as counts (percentages) and compared between groups with Pearson’s χ² test; the assumption of an equal sex ratio was tested with the exact binomial test, and inter-pathogen age differences with the Kruskal–Wallis test. Daily case records were aggregated to monthly totals and merged with the meteorological time series. Yates’ continuity correction was applied to 2 × 2 comparisons.

Co-infections were tabulated separately from the mono-infection pathogen spectrum. The primary pathogen-spectrum analysis (case counts, proportions, and demographic and specimen-type distributions in Section 3.1) attributes each specimen to a single, mutually exclusive pathogen row, so the per-pathogen counts in **Table 1** sum to the total of 2,597 virus-positive cases; co-infection structure itself (pairwise combinations, and multiplicity) is tabulated separately, with each co-infected specimen contributing to every virus it carried, and is described in Section 3.4.

**Table 1:** Demographic and specimen characteristics of pediatric viral LRTI cases by pathogen.

Pathogen	N (% of all positive)	Male, n (%)	Female, n (%)	Sex p-value	Age, median (IQR), y	Lower respiratory tract specimen, n (%)
HRV	796 (30.7)	471 (59.2)	325 (40.8)	<0.001	4.0 (2.0–6.0)	660 (82.9)
ADV	692 (26.6)	425 (61.4)	267 (38.6)	<0.001	6.0 (3.0–8.0)	485 (70.1)
RSV	536 (20.6)	304 (56.7)	232 (43.3)	0.002	3.0 (1.0–4.0)	454 (84.7)
HBoV	313 (12.1)	179 (57.2)	134 (42.8)	0.013	2.0 (1.0–4.0)	277 (88.5)
IFV	155 (6.0)	83 (53.5)	72 (46.5)	0.42	5.0 (2.0–7.0)	123 (79.4)
HMPV	105 (4.0)	66 (62.9)	39 (37.1)	0.011	4.0 (1.8–6.0)	74 (70.5)

Pathogen counts are mutually exclusive per specimen (see Section 2.4), so the column totals sum to 2,597. Co-infected specimens, in which two or more Class A viruses were detected concurrently, are tabulated separately in Section 3.4, where each such specimen contributes to the count of every virus it carried; those pairwise counts are not reflected in the totals above. Sex p-values are from the exact binomial test against a 50:50 null; age is median (IQR) in years; Lower respiratory tract specimen = sputum or BALF.

Pathogen–climate co-variation was screened with Spearman’s rank correlation (ρ) on the full monthly series and re-examined within meteorological seasons (spring, March–May; summer, June–August; autumn, September–November; winter, December–February).

Lagged meteorological effects were examined in two complementary ways. First, cross-correlation functions were computed over lags of 0–6 months for temperature, relative humidity and wind speed. Second, lag-specific relative risks (RR) were derived from quasi-Poisson GLMs with a log link (Bhaskaran et al., 2013; Gasparrini, 2011; Imai et al., 2015) of the form log(*μ*_t_) = α + β × *X*_t−L_, where *μ*_t_ is the expected monthly case count in month *t* and *X*_t−L_ the meteorological exposure shifted back by *L* months (*L* = 0, 1, …, 6). Because this analysis spans 126 model fits (6 pathogens × 3 variables × 7 lags), no formal correction for multiple comparisons was applied; signals reaching p<0.01 (for example, RSV with wind speed at a 1-month lag) are interpreted as relatively robust, whereas signals in the 0.01 to 0.05 range (for example, HMPV with temperature, and RSV with temperature) are flagged as exploratory. RR values are reported per clinically interpretable unit (5 °C for temperature, 10% for relative humidity and 0.5 m/s for wind speed), each chosen to approximate the interquartile span of the corresponding variable. Standard errors were corrected for overdispersion (Pearson dispersion parameter φ ≥ 1). For every pathogen × exposure combination, the lag at which the absolute log-RR was largest among significant lags is reported as the peak RR; the resulting *post-hoc* selection across candidate lags is discussed as a source of potential effect-size inflation in Section 4.6.

This graded reading is a descriptive convention and does not control the family-wise error rate. Under the null hypothesis, roughly six of the 126 fits would be expected to reach p<0.05 by chance, and a Bonferroni-corrected threshold would fall at p<0.0004. We report uncorrected p-values on the reasoning that the six pathogens address distinct epidemiological questions rather than a single universal null, an approach with precedent in exploratory analyses of this kind (Rothman, 1990); the accompanying Type I error risk is set out explicitly in Section 4.6. Two associations in the 0.01 to 0.05 range are nonetheless carried into the Discussion, RSV with temperature and HMPV with temperature, and both are presented there as provisional, the HMPV signal explicitly as hypothesis-generating; no clinical or public health recommendation in this paper rests on either of them alone.

Because the peak RR is the largest of up to seven correlated estimates, it is biased away from the null and its confidence interval is narrower than the selection process warrants, a general property of effect estimates identified by crossing a significance threshold in underpowered analyses (Ioannidis, 2008). The complete lag profile of every pathogen × exposure combination with at least one significant lag is therefore plotted in **Figure 1**, and those lag-specific estimates, rather than the single peak value, should be read as the primary result.

**Figure 1:**
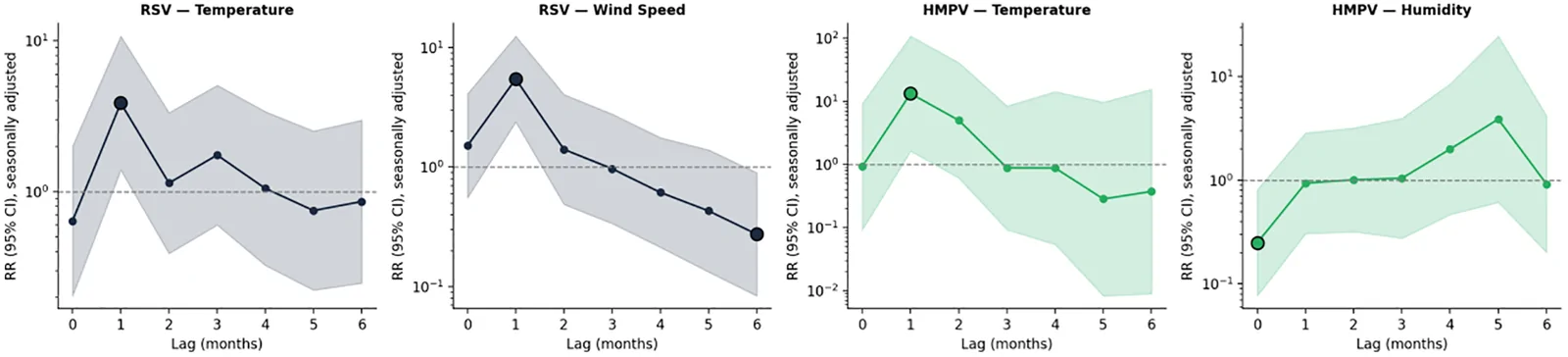
Seasonally- and trend-adjusted relative risk (RR, 95% CI) by lag from the unified quasi-Poisson GLM, for the pathogen × climate variable combinations with at least one significant lag: RSV–temperature, RSV–wind speed, HMPV–temperature and HMPV–humidity. Filled markers with a black outline denote lags reaching p<0.05. The reference RR = 1 is marked by a dashed line; the y-axis is on a log scale.

To address seasonality, secular trend and lagged climate exposure within a single coherent model, we additionally fitted, for each pathogen and each meteorological variable at each lag (0–6 months), a unified quasi-Poisson GLM of the form log(*μ*_t_) = α + β_1_×*X*_t−L_ + β_2_×sin(2π*m*_t_/12) + β_3_×cos(2π*m*_t_/12) + β_4_×*t*_t_. Here *X*_t−L_ is the lagged climate exposure, the sine–cosine pair is a first-order Fourier term that absorbs within-year seasonality, and *t*_t_ is a standardized linear time index that absorbs the secular surveillance-ramp-up trend. In this two-stage design, Spearman correlation serves only as exploratory screening; the single-lag quasi-Poisson model is the core estimator, first fitted without seasonal or trend terms (the “unadjusted” model, which captures the raw temporal phase relationship between a climate exposure and case counts) and then re-fitted with the harmonic seasonal and linear trend terms added (the “unified” model). The two specifications answer distinct questions: the unadjusted model asks whether a lagged climate exposure co-varies with case counts at all, whereas the unified model asks the more stringent question of whether it still predicts case counts once seasonality and secular trend are already accounted for in the same model. Models were fitted by iteratively reweighted least squares with quasi-Poisson (overdispersion-corrected) standard errors.

All numerical work was done in Python 3 (pandas, NumPy, SciPy, matplotlib). A two-sided p<0.05 was the significance threshold throughout.

Co-infection structure was summarized with pairwise co-occurrence matrices, and ranked combination tables. To test whether the most common pair (HBoV+HRV) exceeded chance, an expected co-occurrence frequency was computed under the assumption of independent detection and compared with the observed count using a two-sided Fisher’s exact test.

## 3 Results

### 3.1 Overall pathogen distribution and demographics

Across 36 surveillance months we identified 2,597 virologically confirmed pediatric LRTI cases. HRV was the most frequently detected virus (796 cases, 30.7%), followed by ADV (692, 26.6%), RSV (536, 20.6%), HBoV (313, 12.1%), IFV (155, 6.0%) and HMPV (105, 4.0%) (**Figure 2A**; **Table 1**). Sputum was the predominant specimen type for HRV, RSV, HBoV, HMPV and IFV. The proportion of cases sampled from the lower airway (sputum or BALF) likewise differed across pathogens (χ² = 73.7, df = 5, p < 0.001), ranging from 70.1% for ADV and 70.5% for HMPV to 88.5% for HBoV (**Table 1**). ADV was more evenly distributed across specimen types, with BALF (35.4%) and sputum (34.7%) contributing similar shares and nasopharyngeal swabs a further 29.6%, giving ADV a markedly higher BALF share than the other five viruses (**Figure 2B**). Annual case counts rose from 188 in 2023 to 1,027 in 2024 and 1,382 in 2025 (**Figure 2C**). The 2023 figures warrant particular caution on this account. The assay reached full clinical implementation only in the fourth quarter of that year (Section 2.2), so the 188 cases recorded in 2023 almost certainly under-represent circulation during the early post-pandemic period relative to 2024 and 2025, when testing volume was broadly stable. Subsequent analyses therefore emphasize pathogen composition, age distribution and seasonal timing, which are far less sensitive to testing volume than absolute case counts.

**Figure 2:**
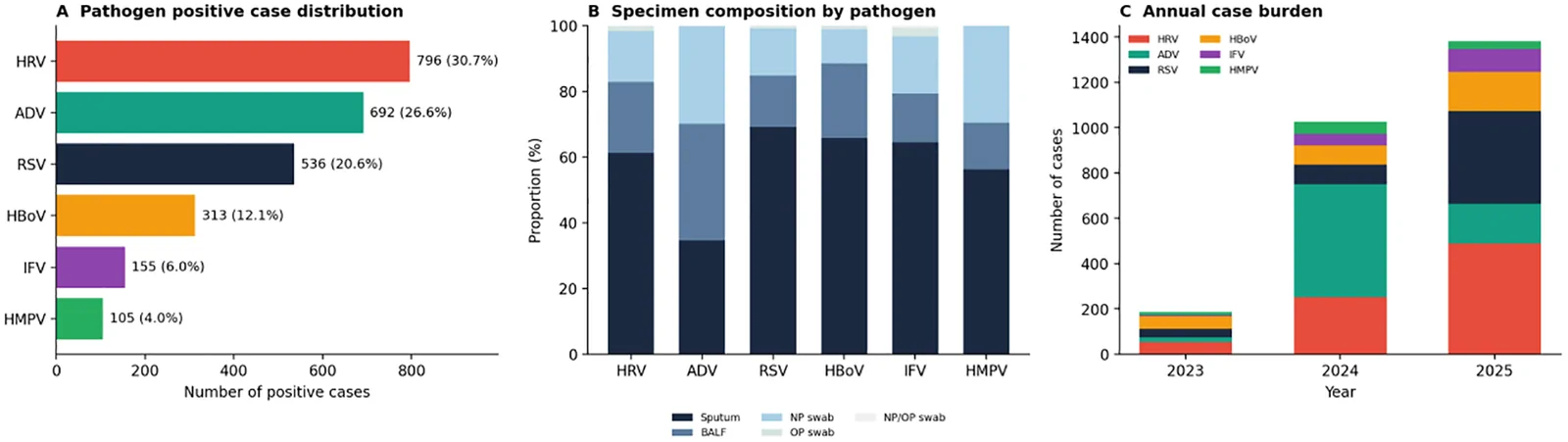
Overall pathogen spectrum and specimen characteristics of pediatric viral LRTI in Tianjin, 2023–2025. **(A)** Case distribution across the six viral pathogens, ranked by prevalence. **(B)** Specimen-type composition by pathogen. **(C)** Annual case burden, stacked by pathogen.

Two features of the assay bound the influence of testing volume without removing it. All six viruses sit on a single multiplex panel and are screened simultaneously in every specimen, so no virus could be tested more often than another, and a change in ordering volume alters which children were tested rather than which viruses were sought. What this cannot exclude is a shift in case mix: if tNGS was ordered preferentially for more severely ill children, and if the pathogen spectrum of severe admissions differs from that of milder ones, composition will move with ordering practice. The markedly higher BALF share of ADV (**Figure 2B**) is consistent with such a gradient, and the point is developed in Section 4.6.

Boys predominated for HRV (59.2%, p<0.001), ADV (61.4%, p<0.001), RSV (56.7%, p=0.002), HBoV (57.2%, p=0.013) and HMPV (62.9%, p=0.011), whereas IFV showed a balanced sex ratio (53.5% male, p=0.42) (**Table 1**; **Figure 3A**). Although IFV was the only virus without a male excess, a χ² test for heterogeneity across the six pathogens did not reach significance (χ² = 5.77, df = 5, p = 0.33), and neither did the comparison of IFV against the other five viruses combined (χ² = 1.68, df = 1, p = 0.20). The balanced ratio for IFV is therefore better read as a failure to demonstrate a male excess in the smallest of the larger groups (n = 155) than as evidence of a genuinely different sex distribution.

**Figure 3:**
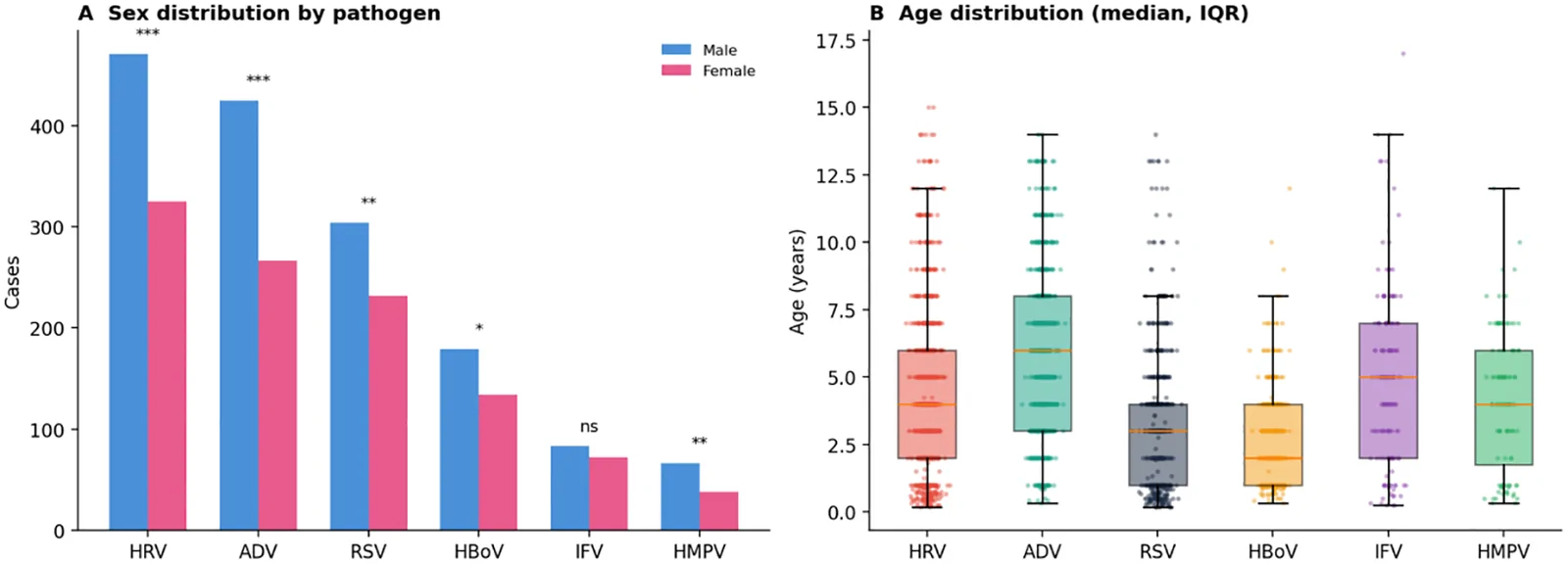
Demographic composition of pediatric viral LRTI by pathogen. **(A)** Sex distribution, with binomial-test significance markers. **(B)** Age (years) by pathogen (median, IQR), with jittered individual data points overlaid. Significance markers: *p<0.05, **p<0.01, ***p<0.001; ns = not significant.

Age distributions differed sharply across pathogens (Kruskal–Wallis H = 372.7, p < 0.001; **Figure 3B**). Infants and toddlers carried most HBoV (median 2.0 [IQR 1.0–4.0] years) and RSV (3.0 [1.0–4.0]) cases; preschoolers were the main group for HRV (4.0 [2.0–6.0]) and HMPV (4.0 [1.8–6.0]); ADV predominated in school-aged children (6.0 [3.0–8.0]), while IFV spanned the older preschool-to-school-age range (median 5.0 [2.0–7.0]) (**Table 1**; **Figure 3B**).

### 3.2 Temporal distribution

Monthly time-series of the six pathogens (**Figure 4A**) showed strong but pathogen-specific seasonality. HRV, ADV, RSV, IFV and HMPV all reached their annual maxima in December, consistent with the cold, dry continental winter being the dominant transmission season in northern China. The December peak of late 2024 (about 400 positive cases that month) was the largest monthly count of the surveillance period, with a smaller but still notable winter peak in December 2025. HBoV behaved differently, showing a single summer maximum in July. ADV showed a secondary peak in late spring (April–May), and HRV and ADV together produced the broadest seasonal envelope (March–December) of any pathogen. The seasonal heatmap (**Figure 4B**) summarizes these patterns: each virus shows at least one clearly defined month-of-year peak, and HBoV is the only virus to peak in a warm-season month (July). Because the heatmap is normalized row-wise, color intensity for each pathogen is scaled to its own monthly range; the visual prominence of a peak for a low-incidence pathogen (for example, HMPV) therefore does not indicate the same absolute case count as for a high-incidence pathogen (for example, HRV).

**Figure 4:**
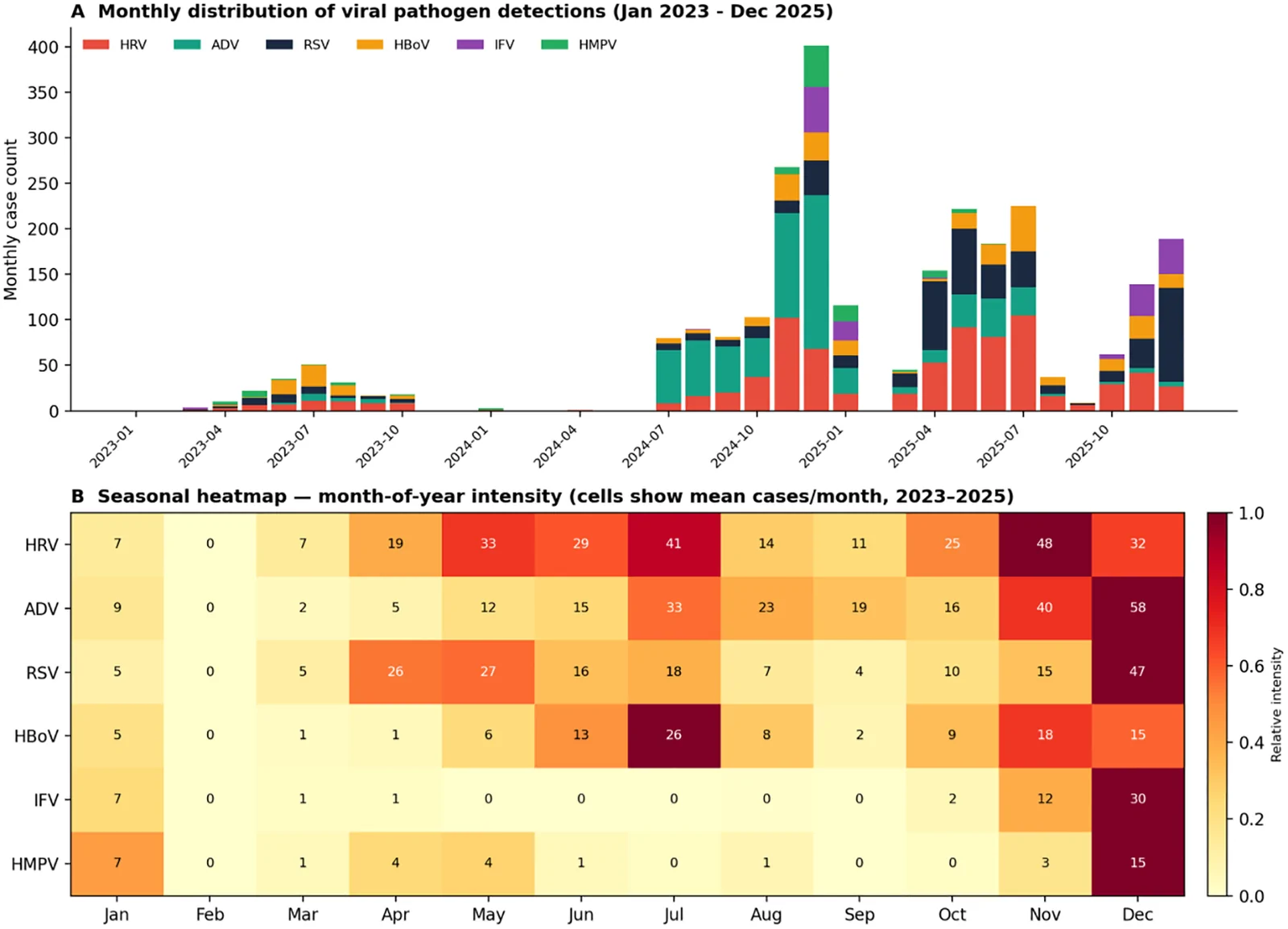
Temporal distribution of pediatric viral LRTI, 2023–2025. **(A)** Stacked monthly case counts by pathogen. **(B)** Seasonal heatmap of mean monthly cases by pathogen; cell shading is normalized row-wise so that each pathogen’s twelve-month profile can be read on a comparable scale.

### 3.3 Seasonal and meteorological analyses

#### 3.3.1 Overall pathogen–climate correlations

Overall Spearman rank correlations (**Figure 5A**; **Table 2**) showed a significant positive correlation between HBoV and temperature (ρ = +0.34, p = 0.04). ADV (ρ = +0.32, p = 0.06), HRV (ρ = +0.28, p = 0.10) and RSV (ρ = +0.24, p = 0.16) showed positive but non-significant trends. IFV and HMPV showed non-significant negative correlations with temperature (ρ = −0.27 and −0.05). Relative humidity followed a similar pattern: HBoV (+0.33), ADV (+0.30) and HRV (+0.28) correlated positively, whereas IFV (−0.29) and HMPV (−0.23) correlated negatively. Wind speed produced predominantly negative correlations across the six viruses, strongest for HBoV (−0.32), ADV (−0.30) and HRV (−0.29).

**Figure 5:**
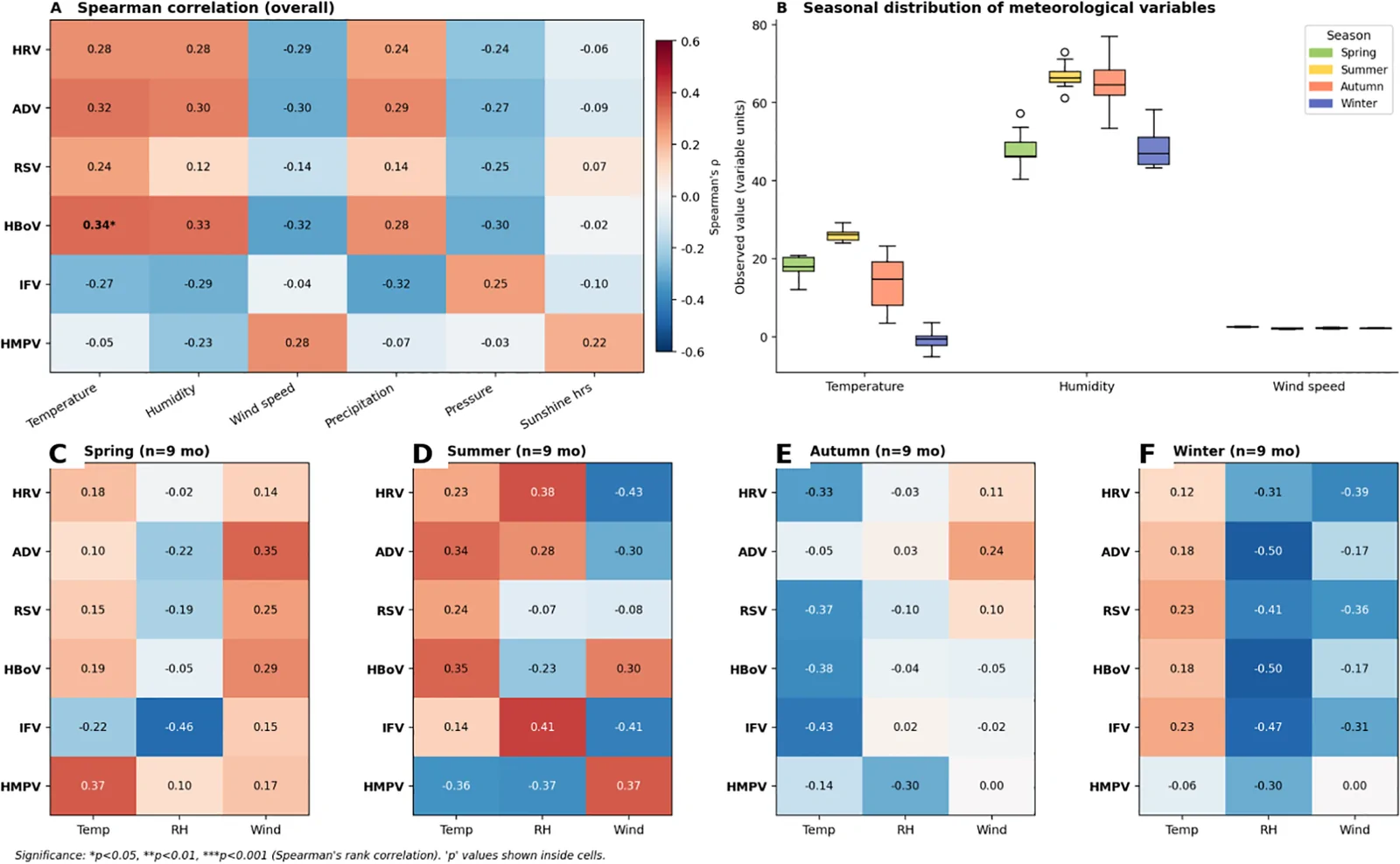
Pathogen–meteorology correlations. **(A)** Overall Spearman ρ between monthly case counts and six meteorological variables; the color bar is fixed at the symmetric range −1 to +1 to allow direct comparison across panels. **(B)** Seasonal distribution of temperature, relative humidity and wind speed. **(C–F)** Season-stratified Spearman ρ for the three core exposures across spring, summer, autumn and winter, using the same −1 to +1 symmetric scale. Cell labels show ρ with significance markers (*p<0.05, **p<0.01, ***p<0.001).

**Table 2:** Overall Spearman rank correlations between monthly viral case counts and city-level meteorological indicators in Tianjin, 2023–2025.

Pathogen	Temperature (°C)	Relative humidity (%)	Wind speed (m/s)	Precipitation (mm)	Pressure (hPa)	Sunshine duration (h)
HRV	+0.28	+0.28	−0.29	+0.24	−0.24	−0.06
ADV	+0.32	+0.30	−0.30	+0.29	−0.27	−0.09
RSV	+0.24	+0.12	−0.14	+0.14	−0.25	+0.07
HBoV	+0.34*	+0.33	−0.32	+0.28	−0.30	−0.02
IFV	−0.27	−0.29	−0.04	−0.32	+0.25	−0.10
HMPV	−0.05	−0.23	+0.28	−0.07	−0.03	+0.22

*p<0.05; values without a marker are non-significant (p>0.05).

#### 3.3.2 Season-stratified correlations

Season-stratified analyses (**Figures 5C–F**), set against the seasonal distribution of the weather variables themselves (**Figure 5B**), refined these patterns, although all season-stratified correlations should be regarded as exploratory and hypothesis-generating rather than confirmatory given the small number of months per season (n = 9). In spring, IFV showed a negative correlation with relative humidity (ρ = −0.46), consistent with residual influenza transmission under cold, dry conditions after the main winter wave (**Figure 5C**). In summer, IFV and HRV showed positive humidity correlations (ρ = +0.41 and +0.38), and HMPV showed negative correlations with both temperature (−0.36) and humidity (−0.37) (**Figure 5D**). In autumn, HBoV and IFV showed negative correlations with temperature (−0.38 and −0.43), capturing the cool end of their transmission windows (**Figure 5E**). In winter, IFV, ADV and HBoV all showed negative correlations with relative humidity (ρ from −0.47 to −0.50), consistent with cold, dry air as a potential environmental factor during the December–February peak, though causality cannot be inferred from these exploratory correlations (**Figure 5F**). The season-dependent reversal of correlation direction, for example HRV’s humidity correlation of +0.38 in summer versus −0.31 in winter, underlines the need for seasonal stratification when interpreting climate–pathogen relationships (Chen et al., 2014a; Cui et al., 2015; Xu et al., 2021).

#### 3.3.3 Lagged climate effects (unadjusted analysis)

Cross-correlation functions over 0–6-month lags (**Figure 6**) revealed distinct pathogen-specific lag profiles. For temperature, positive correlations peaked at the 1– to 2-month lag for HBoV (ρ = +0.40 at lag 1) and ADV (ρ = +0.39 at lag 1, + 0.37 at lag 2), with HRV also positive across short lags; IFV showed strongest temperature correlations at longer lags (IFV ρ = +0.38 to +0.40 at lags 4–5), consistent with cold-season activity carrying into the following spring. Relative humidity behaved similarly, with positive associations for HBoV, ADV and HRV at lags 0–2 months and positive associations for IFV at lags 4–5 months (ρ = +0.37 to +0.39). Wind speed produced predominantly negative cross-correlations for HRV and ADV at lag 0 (ρ = −0.29 and −0.30), while IFV showed a significant negative wind-speed correlation at lags 2–3 months (ρ = −0.44 and −0.42).

**Figure 6:**
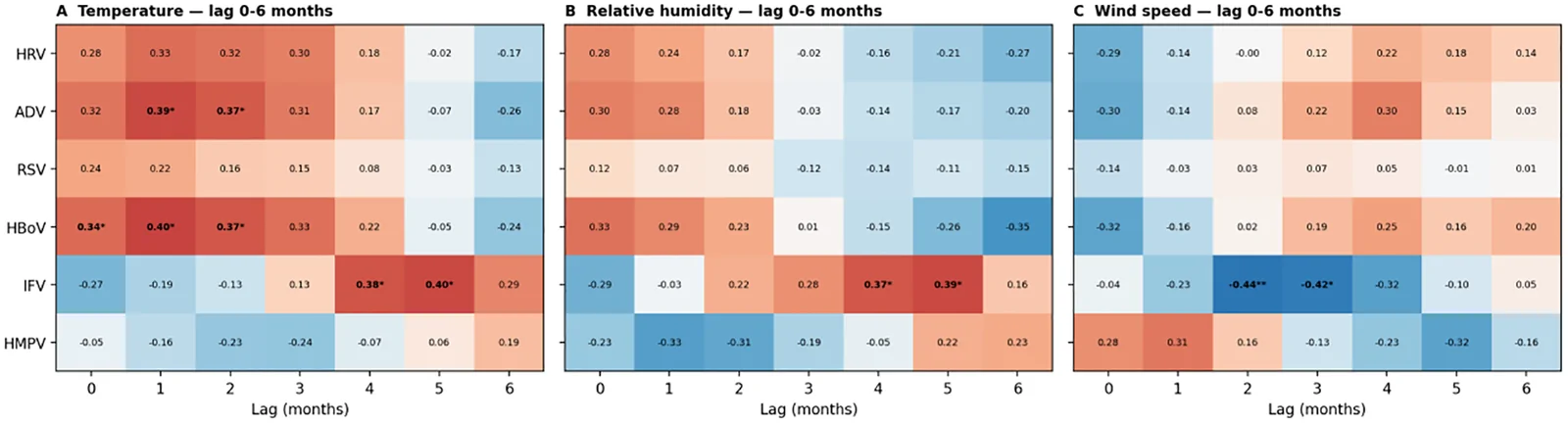
Cross-correlation of monthly case counts with lagged meteorological exposures, 0–6-month lags (Spearman ρ). **(A)** Temperature. **(B)** Relative humidity. **(C)** Wind speed. The color bar uses a symmetric −1 to +1 scale; cell labels show ρ with significance markers (*p<0.05, **p<0.01; no cells reached ***p<0.001).

Lag-specific quasi-Poisson RR estimates from models that did not adjust for seasonality are shown in **Table 3**. These RRs quantify the temporal phase relationship (sub-annual synchrony) between a lagged climate variable and case counts, not a direct causal effect. The most pronounced synchrony was for IFV: a 10% increase in relative humidity at a 3-month lag corresponded to a 3.58-fold higher case count (95% CI 1.68–7.60, p<0.001). HMPV also reached significance in this unadjusted analysis (**Table 3**). For HRV, ADV, RSV and HBoV, no lagged association reached significance in the unadjusted analysis (all p>0.05).

**Table 3:** Significant lag-specific quasi-Poisson GLM relative risks (per standard unit of meteorological exposure) for IFV and HMPV, at the peak lag within the 0–6-month window (unadjusted analysis; p<0.05).

Pathogen	Meteorological variable	Unit	Lag (months)	Peak RR (95% CI)	p
IFV	Temperature	per 5 °C	4	3.52 (1.30–9.53)*	0.013
IFV	Relative humidity	per 10%	3	3.58 (1.68–7.60)***	<0.001
IFV	Wind speed	per 0.5 m/s	3	0.06 (0.01–0.25)***^†^	<0.001
HMPV	Temperature	per 5 °C	6	1.74 (1.05–2.88)*	0.033
HMPV	Wind speed	per 0.5 m/s	4	0.28 (0.09–0.86)*	0.026

RR, relative risk; CI, confidence interval. Significance markers: *p<0.05; ***p<0.001 (no estimates in this table fell in the **p<0.01 range). ^†^The IFV wind-speed estimate at lag 3 (RR 0.06) is statistically unstable given the small case number (n = 155) and collinearity among climate variables; it is reported for completeness but is not interpreted substantively and should not be read as evidence of a protective wind-speed effect.

#### 3.3.4 Unified seasonally- and trend-adjusted model

To assess whether lagged climate exposures predict case counts once seasonality and secular trend are simultaneously accounted for in a single model, we fitted a unified quasi-Poisson GLM per pathogen × climate variable × lag (126 models in total; Section 2.4), incorporating a harmonic seasonal term and a linear time trend alongside the lagged climate exposure.

This baseline model, containing the seasonal-plus-trend terms alone with no climate variables, explained a substantial share of each pathogen’s monthly deviance: 77.5% for IFV, 59.6% for RSV, 41.5% for HRV, 28.6% for HBoV, 20.3% for HMPV and 17.1% for ADV (**Table 4**). This confirms that seasonality is the dominant structure in all six time-series and that any climate association must be evaluated against this strong seasonal baseline.

**Table 4:** Baseline model performance: percentage of monthly deviance explained by seasonal (harmonic) and secular (linear time) terms alone, before adding any lagged climate exposure.

Pathogen	Deviance explained by season + trend (%)
IFV	77.5
RSV	59.6
HRV	41.5
HBoV	28.6
HMPV	20.3
ADV	17.1

This baseline model contains no climate variables and is distinct from the unified model (**Table 5**), which adds lagged climate exposure to the same seasonal and trend terms.

Values are the percentage of monthly deviance explained by the harmonic seasonal term plus linear time trend, before any lagged climate exposure is added, and index the strength of each pathogen’s intrinsic seasonal structure.

Once seasonality and trend were held constant within the same model, the pattern of significant lagged climate associations changed substantially from the unadjusted analysis (**Figure 1**; **Table 5**). IFV’s previously prominent lag-3 humidity and lag-4 temperature signals were no longer significant in the adjusted model (humidity at lag 3: RR 0.91, 95% CI 0.13–6.33, p=0.92; temperature at lag 4: unstable and non-significant), consistent with the interpretation that the apparent climate associations for IFV are largely a reflection of its strong intrinsic seasonality (77.5% of deviance explained by season and trend alone), and that any independent climate effect, if present, may be below the detection limit of our model given the sample size (n = 155). Conversely, two associations emerged as significant in the unified model that had not reached significance in the unadjusted analysis. RSV showed a significant positive association with temperature at a 1-month lag (RR 3.87, 95% CI 1.39–10.76, p=0.014) and with wind speed at a 1-month lag (RR 5.46, 95% CI 2.39–12.47, p<0.001), with a weaker inverse association at a 6-month lag (RR 0.27, 95% CI 0.08–0.90, p=0.043). HMPV retained a significant association with temperature at a 1-month lag (RR 13.3, 95% CI 1.64–108.3, p=0.022) and showed a new inverse association with humidity at lag 0 (RR 0.25, 95% CI 0.08–0.81, p=0.028). HRV, ADV and HBoV showed no significant lagged climate association in the unified model (all p>0.05).

**Table 5:** Significant lagged climate associations after seasonal and trend adjustment.

Pathogen	Climate variable	Lag (months)	Unit	RR (95% CI)	p
RSV	Temperature	1	per 5 °C	3.87 (1.39–10.76)	0.014
RSV	Wind speed	1	per 0.5 m/s	5.46 (2.39–12.47)	<0.001
RSV	Wind speed	6	per 0.5 m/s	0.27 (0.08–0.90)	0.043
HMPV	Temperature	1	per 5 °C	13.33 (1.64–108.29)	0.022
HMPV	Relative humidity	0	per 10%	0.25 (0.08–0.81)	0.028

HRV, ADV and HBoV showed no significant lagged climate association after adjustment (all p>0.05). IFV also showed no significant adjusted association, in contrast to its prominent unadjusted signal (**Table 3**; Section 3.3.3), consistent with IFV’s associations reflecting shared seasonality (77.5% of deviance explained by season and trend alone) rather than an independent climate effect.

### 3.4 Co-infection structure

HRV was the most frequently co-detected pathogen (80/116 events, 69.0%), followed by HBoV (55.2%), RSV (48.3%), IFV (25.9%), ADV (14.7%) and HMPV (3.4%) (**Figure 7B**). The leading combinations were HBoV+HRV (27 cases, 23.3%), HRV+RSV (18, 15.5%), HBoV+RSV (15, 12.9%), HRV+IFV (11, 9.5%) and IFV+RSV (9, 7.8%) (**Figure 7A**); among co-infected cases, 83.6% involved two pathogens and 16.4% three, with HBoV+HRV+RSV (8 cases) the most frequent triple combination (**Figure 7C**). To test whether the leading HBoV+HRV pairing exceeded the frequency expected under independent detection, we compared the observed count with the expected co-occurrence frequency using a two-sided Fisher’s exact test. Contrary to the *a priori* expectation, HBoV+HRV co-detection occurred significantly less often than predicted under independent detection (expected n = 95.9; observed n = 27; OR = 0.19, 95% CI 0.12–0.28; p < 0.001, two-sided Fisher’s exact test). Co-infections clustered within the case peaks of mid-to-late 2024 and late 2025 (**Figure 7D**).

**Figure 7:**
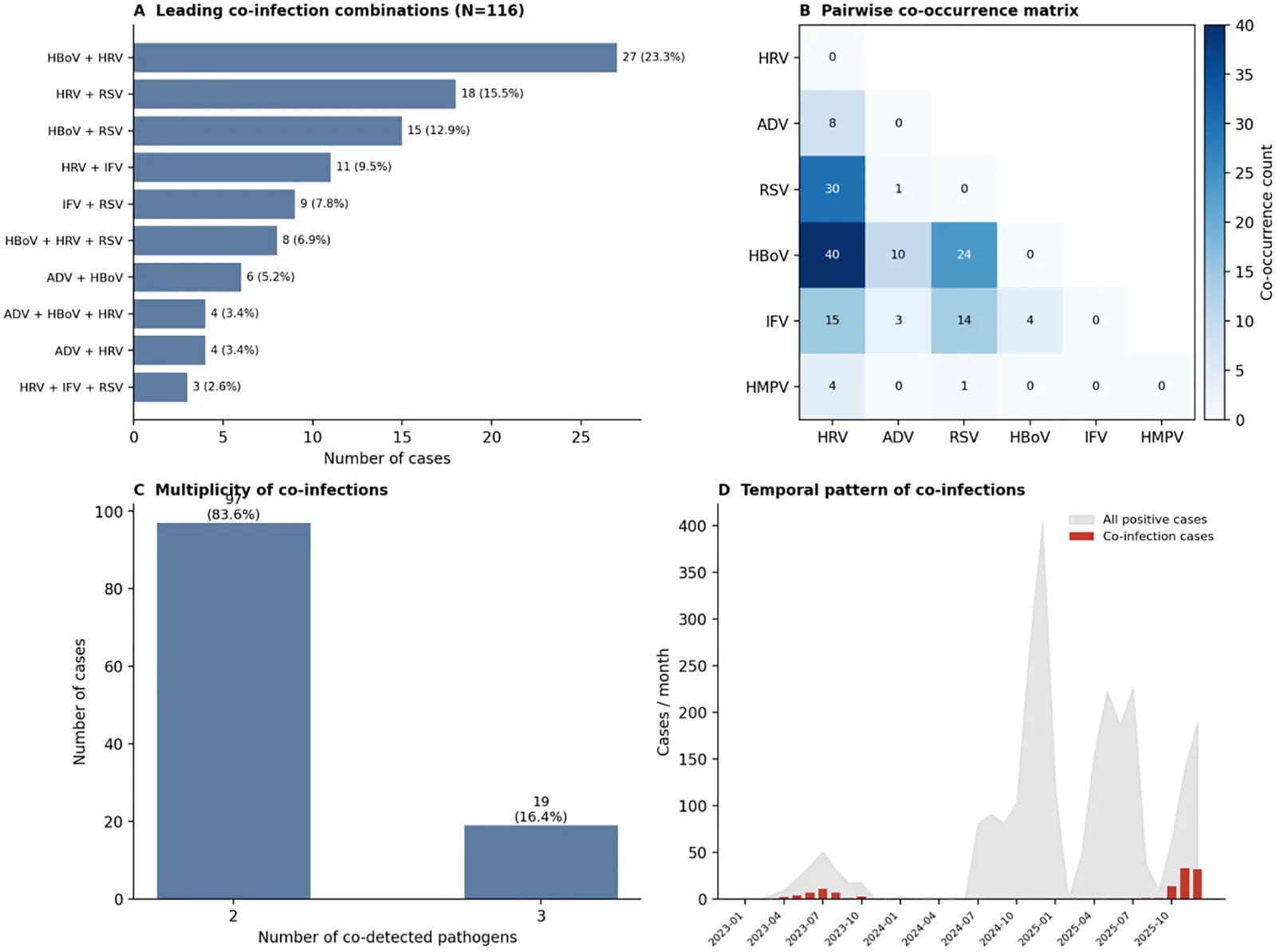
Co-infection structure of pediatric viral LRTI in Tianjin, 2023–2025 (N = 116 co-infection events). **(A)** Leading pathogen combinations. **(B)** Pairwise co-occurrence matrix (lower triangular). **(C)** Multiplicity of co-infections (two versus three pathogens). **(D)** Monthly distribution of co-infection cases overlaid on the total positive-case envelope.

## 4 Discussion

In this three-year, single-center cohort we characterized the post-pandemic epidemiology of pediatric viral LRTI in Tianjin, a megacity on the North China Plain, and quantified how closely monthly viral activity tracks ambient weather. To our knowledge, the dataset is unusual for northern China in spanning six respiratory viruses, including the comparatively under-surveyed HRV, HBoV and HMPV, over the full 2023–2025 post-pandemic rebound window, with a complete and time-matched monthly meteorological record.

### 4.1 Principal epidemiological features

Across the six viruses, HRV, ADV and RSV were the leading causes of pediatric LRTI, and each pathogen carried a well-defined demographic profile. Age and sex stratification confirmed recognized biological gradients. RSV and HBoV were most prevalent in infants and toddlers, in line with airway and immune development (Feng et al., 2024; Jiang et al., 2016; Li et al., 2022); ADV peaked in school-aged children and IFV in the older preschool-to-school-age range, consistent with close-contact transmission in schools and other crowded settings (Fang et al., 2025; Yu et al., 2021; Zhang et al., 2023); and HMPV showed an intermediate distribution. The older age skew of IFV fits its epidemiology in temperate regions, where school-aged children are the principal drivers of seasonal influenza transmission and the group in which symptomatic infection is most readily recognized, in contrast to the infant and toddler predominance of RSV and HBoV (Feng et al., 2024; Klein and Flanagan, 2016). The male predominance for HRV, ADV, RSV, HBoV and HMPV, but not IFV, echoes prior pediatric LRTI cohorts and may reflect sex differences in innate immunity and behavior (Klein and Flanagan, 2016). The strong age-group × pathogen association reinforces the need for an explicitly age-stratified surveillance framework.

The six pathogens also fell into two distinct seasonal groups. HRV, ADV, RSV, IFV and HMPV all peaked in December, during the cold, dry continental winter, whereas HBoV stood apart with an isolated July maximum—the only warm-season peak of the panel. HRV additionally displayed the broadest seasonal envelope, circulating from March to December, consistent with fundamentally endemic, weakly seasonal behavior. Because the rise in absolute case counts partly reflects expanding testing volume rather than incidence (Section 3.1), the interpretation that follows rests on pathogen composition, age structure and seasonal timing rather than on raw counts.

### 4.2 Interpretation in the post-pandemic context

These patterns are best read against, rather than directly measured against, pre-pandemic Chinese pediatric surveillance, since our cohort describes the post-pandemic period without a matched pre-pandemic Tianjin comparator. Several multi-center Chinese studies contrasting pre-pandemic (2018–2019) with post-pandemic (2023 onward) cohorts have reported a broadly consistent post-pandemic pattern: higher overall pathogen positivity and mixed-infection rates, a higher proportion of severe presentations, and shifts in the relative ranking, seasonal timing and age distribution of individual pathogens (Cui et al., 2024; Liu et al., 2025). In Shanghai, post-pandemic test positivity rose markedly relative to 2019, with both RSV and ADV increasing and RSV’s onset shifting earlier into August, ahead of its conventional winter season (Liu et al., 2025); other Chinese cohorts spanning the pre-pandemic, pandemic and post-pandemic phases have likewise documented altered seasonal timing and age-distribution shifts for RSV, ADV and rhinovirus after non-pharmaceutical interventions were relaxed (Cui et al., 2024).

Interpreted against this backdrop, our Tianjin observations show both continuities and departures. HRV’s endemic, weakly seasonal circulation—reflected in its leading position and broad March–December envelope—appears to have been less perturbed by the pandemic and its aftermath than that of the more strongly seasonal viruses. By contrast, the convergence of five of the six viruses onto a single sharp December peak is consistent with the compressed, intensified post-pandemic winter surge described elsewhere in China, plausibly reflecting the accumulated immunity debt from population-level exposure gaps in 2020–2022 (Cohen et al., 2021; Li et al., 2024) superimposed on the resumption of normal contact patterns. HBoV departed most sharply from this winter pattern, peaking instead in summer; to our knowledge, such an isolated warm-season peak has not been prominently reported as a post-pandemic phenomenon elsewhere in China. Whether it reflects a genuine post-pandemic shift in HBoV’s seasonal niche or a feature specific to Tianjin’s continental climate cannot be resolved with a single-center, single-period dataset, and we return to its biological and clinical significance in Section 4.4.

### 4.3 Climate-driven lagged effects

The unadjusted and seasonally adjusted analyses answer different questions, and reading them together guards against a common pitfall in climate–infection studies. In the unadjusted analysis (**Table 3**), IFV appeared the most climate-responsive virus, with antecedent humidity and temperature aligning with later case counts, in keeping with the recognized seasonal phasing of influenza in temperate climates (Deyle et al., 2016; Kudo et al., 2019; Lowen et al., 2007; Lowen and Steel, 2014). Once seasonality and secular trend were held constant in the unified model, however, these IFV associations disappeared (**Table 5**). This does not mean that IFV has no climatic drivers. Rather, because ambient temperature and humidity in Tianjin themselves follow an almost perfect annual sinusoid, they are nearly collinear with the sine–cosine seasonal terms of the unified model; for a virus as tightly locked to the annual cycle as influenza (77.5% of deviance explained by season and trend alone), the model simply cannot separate the effect of “season” from that of “temperature” or “humidity.” The most that can be concluded is that, at the present monthly resolution, IFV shows no climatic association detectable independently of the seasonal cycle itself.

The seasonally adjusted model instead isolated RSV and HMPV as the only viruses with climate associations independent of shared seasonality (**Table 5**): RSV tracked temperature and wind speed at short lags, while HMPV tracked temperature positively and humidity inversely, although its low case counts make these estimates imprecise and hypothesis-generating. HRV, ADV and HBoV showed no independent lagged signal, indicating that at monthly resolution their seasonal envelopes are not accompanied by a detectable climate effect beyond the annual cycle. This null result should not be read as evidence of climatic insensitivity. For these three viruses the seasonal-plus-trend baseline itself explained comparatively little of the monthly deviance (HRV 41.5%, HBoV 28.6%, ADV 17.1%; **Table 4**), so a large share of the variation in their case counts is left unaccounted for by season, trend and weather combined. Residual variation of that magnitude widens the standard errors, and with 36 monthly observations spanning three seasonal cycles only associations of substantial size could reach significance. A modest but real climate effect would probably have escaped detection here, and the finding is better described as uninformative for HRV, ADV and HBoV than as negative. The contrast between the two analyses carries the key methodological lesson: lagged climate associations in surveillance data can be interpreted only after seasonality has been accounted for.

These post-pandemic patterns can be read against the pre-pandemic national baseline of Xu et al. (2021), who modelled a seven-virus panel—five of them shared with our own—across 81 sentinel hospitals in 22 Chinese provinces (2009–2013) and ranked the meteorological factors by climate region. For the temperate north, to which Tianjin belongs, three points agree with our data: temperature was among the most explanatory factors for most viruses, matching the temperature associations that survived seasonal adjustment here for RSV and HMPV; relative humidity was dominant specifically in the north but not the south, consistent with the humidity signals we observed for HMPV and, before adjustment, for IFV; and RSV, influenza and HMPV all peaked in winter or winter–spring in the north, mirroring the December convergence of five viruses in our cohort. Our analysis adds to that pre-pandemic, correlation-based picture by separating lagged climate effects from shared seasonality within a single model, showing that some seemingly climate-driven signals—most clearly for IFV—are in fact seasonal artefacts.

Two mechanisms sit behind the associations that did survive adjustment, and both are governed by the variables we modelled. The first is aerosol behavior. Exhaled droplets evaporate rapidly at low relative humidity to form droplet nuclei that stay airborne longer and travel further, and the survival of enveloped viruses within those particles depends jointly on temperature and relative humidity rather than on absolute humidity alone (Marr et al., 2019; Moriyama et al., 2020). Wind speed acts on the same physical process outdoors, and indirectly indoors through ventilation and infiltration. The second is host susceptibility. Cold, dry inspired air impairs mucociliary clearance and barrier function in the airway epithelium and blunts interferon-mediated antiviral responses, so a given inoculum is more likely to establish infection under winter conditions than under summer ones; the temperature dependence of this response has been demonstrated directly for rhinovirus in airway epithelial cells (Foxman et al., 2015; Kudo et al., 2019; Moriyama et al., 2020). Neither aerosol behavior nor host susceptibility was measured in our data, so these pathways are offered as compatible with the direction of the RSV and HMPV associations rather than as demonstrated mechanisms.

### 4.4 HBoV’s summer peak and co-infection

The isolated July epidemic of HBoV, the only warm-season peak among the six viruses (Section 3.2), is the most distinctive feature of the cohort. Its single-stranded DNA genome and non-enveloped capsid confer greater environmental stability than the lipid envelopes of RSV, influenza or HMPV (Chen et al., 2014b; Jiang et al., 2016; Lion, 2014), plausibly allowing it to circulate in summer when enveloped viruses are suppressed by heat and humidity; summer day-care attendance, air-conditioned indoor settings, and incidental respiratory screening of children admitted for other summer illnesses (for example, hand-foot-mouth disease) may contribute further.

The comparison with earlier work is worth setting out in detail, since it bears on whether the July peak is a post-pandemic change. Xu et al. (2021) placed national HBoV positivity in the spring and summer months, and city-level studies converge on the same warm-season pattern with some variation in its timing: in Suzhou, activity ran from June to November with a summer maximum (Chen et al., 2014b); in Changsha, summer accounted for 32.2% of detections (Zhou et al., 2018); in Guangzhou, positivity peaked in May and June (Liu et al., 2011). Northern and northwestern cohorts are less uniform, with an autumn–winter predominance reported from Ningxia (Ji et al., 2021). The Tianjin maximum therefore falls at the later edge of the national spring–summer window rather than outside it, and is close in timing to the eastern and central Chinese cohorts. Post-pandemic data point the same way: HBoV circulating in Beijing during 2023 was likewise more prevalent in the summer months (Lv et al., 2025). Taken together, the evidence favors continuity with pre-pandemic seasonality rather than a post-pandemic shift, and what distinguishes our cohort is the narrowness of the peak, a single month rather than a warm-season plateau, seen against a winter-dominant background for the other five viruses. Confirming that the narrowing is real would require a matched pre-pandemic Tianjin cohort, which we did not have.

Co-infection was uncommon (4.5% of positives). Although HBoV+HRV was the most frequent specific pair in absolute terms, it occurred significantly less often than expected under independent detection (Section 3.4: observed 27 vs. expected 95.9; OR 0.19, 95% CI 0.12–0.28; p < 0.001). This shortfall argues against a facilitative HBoV–HRV interaction and instead suggests competitive viral interference or a detection ceiling in the tNGS workflow when one virus is present at high titer; distinguishing these possibilities would require paired viral-load data. Because both viruses are highly prevalent, HBoV+HRV nonetheless remains the most common co-detection and a reasonable target for clinical vigilance, and the possibility that HBoV is at times a co-pathogen or bystander rather than the primary cause merits confirmation by quantitative PCR or serology (Wang et al., 2022; Zhu et al., 2020); whether such co-infection aggravates disease relative to mono-infection remains unresolved and warrants prospective study (Goka et al., 2015; Li et al., 2020; Mansbach et al., 2012; Patel et al., 2022; Schildgen et al., 2008).

### 4.5 Clinical and public health implications

The combined pathogen, age, seasonal and specimen profile has direct value for diagnosis and treatment. In a febrile infant admitted in late autumn or winter, RSV remains the most likely cause, and nirsevimab-eligible infants should be identified for follow-up (Assad et al., 2024; Drysdale et al., 2023; Hammitt et al., 2022; Moline et al., 2024). In school-aged children with consolidative pneumonia, ADV deserves consideration alongside Mycoplasma pneumoniae, and its comparatively high BALF yield means bronchoscopic sampling can be informative when ADV is suspected and bronchoscopy is otherwise clinically indicated (Fang et al., 2025; Zhang et al., 2023). In preschoolers with persistent wheeze, HRV should be actively sought, given its link to both acute admission and later asthma (Bizot et al., 2021; Costa et al., 2014; Royston and Tapparel, 2016). During June and July, the summer HBoV peak makes this virus an important differential diagnosis in northern China. The year-round dominance of viral over bacterial causes argues against empirical antibiotics in stable children with a confirmed viral mono-infection (Brendish et al., 2017; Hersh et al., 2013; Royston and Tapparel, 2016).

For preparedness, as distinct from real-time triage, the seasonally adjusted results change how climate signals should be used. Because IFV’s apparent climate response is an artefact of seasonality, influenza planning is better anchored to the established December peak and national surveillance than to any antecedent humidity threshold (Grohskopf et al., 2024; Lowen and Steel, 2014); the RSV temperature and wind-speed associations offer only a short lead time and should complement, not replace, local case surveillance (Assad et al., 2024; Moline et al., 2024). How useful that lead time is depends on the decision it feeds. One month is too short for interventions that run on procurement and logistics cycles of several months, such as influenza vaccine-campaign planning or nirsevimab purchasing, and it should not be used to set them. It is better matched to shorter-horizon operational decisions, ward-staffing rosters, oxygen supply and isolation-bed capacity among them, for which a two-to-four-week forecast window is actionable. For HMPV the case is weaker still: the temperature association rests on 105 cases with a confidence interval spanning nearly two orders of magnitude, so we would not recommend planning against it until it has been replicated in a larger series. In operational terms, the December convergence and the secondary July HBoV peak provide a concrete calendar for ward staffing, oxygen and isolation capacity, RSV passive immunization and influenza vaccination timing (Drysdale et al., 2023; Hammitt et al., 2022; Simões et al., 2025). More broadly, this cohort contributes a reference point from the temperate continental climate of northern China to a post-pandemic surveillance literature still dominated by subtropical southern cities (Cui et al., 2015; Fang et al., 2025; Li et al., 2025; Wang et al., 2022), and the single quasi-Poisson framework that fits seasonality, trend and lagged climate together offers a tractable template for other monthly-resolution networks. These implications remain provisional: derived from an exploratory, single-center, monthly-resolution analysis, they would need replication with higher-resolution, denominator-based data before they could inform operational surveillance calendars. The 2023–2025 window nonetheless provides a baseline for future work, including a within-Tianjin pre- versus post-pandemic comparison and prospective evaluation of nirsevimab and maternal RSV vaccination (Baker et al., 2019; Cohen et al., 2021; Li et al., 2024).

### 4.6 Limitations

Several constraints temper these findings. Case ascertainment is imperfect: the tNGS Class A flag is a qualitative confidence label rather than a uniform abundance threshold, so incidental detections of viruses prone to subclinical carriage—particularly HRV and HBoV—cannot be fully excluded and may inflate their apparent contribution. The scale of this problem is illustrated by comparisons with asymptomatic controls, in which influenza, RSV and HMPV are detected almost exclusively in children with pneumonia, whereas HRV is recovered appreciably often from healthy children, and HBoV-1 DNA can persist in the nasopharynx for weeks after an acute episode (Martin et al., 2015; Self et al., 2016). Requiring a clinical LRTI diagnosis for cohort entry, together with the additional adjudication applied to HRV and HBoV (Section 2.1), reduces but cannot eliminate this risk.

A second set of constraints concerns who was tested. As the only tertiary-A comprehensive children’s hospital in Tianjin, with a high proportion of lower-airway specimens, the cohort is enriched for severe and referred disease, and because no citywide denominator of tested or hospitalized cases exists, positivity could not be modelled in place of raw counts. We also could not establish what clinical thresholds prompted a clinician to order tNGS; if the assay was requested preferentially for more severely ill children, pathogens with different severity profiles—ADV and RSV, for instance—would be affected unequally. Emphasizing composition, age distribution and seasonal timing rather than absolute counts (Section 3.1) limits, but does not remove, a bias that operates on which children were tested. The incomplete rollout of testing in 2023 (Section 2.2) compounds this: that year contributes fewer months of substantial case counts to the time-series models than 2024 or 2025, so the lag estimates are weighted toward the later two years, though no inference in this paper otherwise rests on the year-on-year rise.

Specimen type is a further, uneven source of detection bias. ADV was distributed much more evenly across specimen types than the other five viruses, with BALF accounting for 35.4% of its cases against sputum 34.7% and nasopharyngeal swab 29.6%. Because BALF is obtained at bronchoscopy, a procedure reserved for lobar consolidation, atelectasis or non-resolving illness, ADV detections are drawn disproportionately from more severely affected and older children, so its share of the pathogen spectrum and its age profile here are probably higher than a cohort sampled only from the upper airway would show. The reverse consideration applies to the sputum-predominant viruses, where yield may partly reflect which children could produce a specimen rather than true lower-airway tropism; without severity indices, these possibilities cannot be separated.

The meteorological and statistical modeling is further constrained by resolution and sample size. Only monthly weather data were available, so the lag-specific relative risks describe sub-annual phase relationships rather than short-lag (1–2-week) biophysical mechanisms and should not be read as causal; a weekly-resolution, distributed-lag analysis is an important next step (Gasparrini et al., 2010; Li et al., 2025). Of the 126 model fits, roughly six associations would be expected to reach p < 0.05 by chance alone (Section 2.4), and five did so in the unified model, so this set cannot be distinguished from chance by number alone; only RSV with wind speed at a 1-month lag (p < 0.001) approaches the Bonferroni threshold of p < 0.0004. We accordingly treat the RSV associations as the more robust and the HMPV associations—resting on only 105 cases, with intervals spanning nearly two orders of magnitude—as exploratory and hypothesis-generating, and direct readers to the full lag profiles in **Figure 1** rather than to the peak RR values, which are liable to selection-driven inflation (Section 2.4). Power was limited for the low-incidence IFV and HMPV, these signals require independent replication, ideally with negative-binomial or penalized-likelihood models, and IFV was analyzed without influenza A/B subtyping.

Finally, the single-center, single-period design and the absence of a matched pre-pandemic Tianjin cohort mean that post-pandemic change is inferred from other Chinese cities rather than demonstrated here; only three seasonal cycles were observed, and clinical severity, bacterial co-infection and air-pollutant data were unavailable. Multi-center cohorts with weekly- or daily-resolution meteorology, denominator-based metrics, individual-level age adjustment and paired pre- and post-pandemic data will be needed to consolidate these patterns.

## 5 Conclusion

Across 36 months of post-pandemic surveillance in Tianjin (2,597 confirmed cases), HRV, ADV and RSV were the leading viral causes of pediatric LRTI, and each of the six viruses carried a distinctive age, sex, seasonal and specimen-type profile. In a unified model adjusting simultaneously for seasonality and secular trend, RSV and HMPV, rather than IFV, showed the clearest evidence of an independent, non-seasonal climate association, whereas IFV’s prominent unadjusted climate signal proved to reflect its strong intrinsic seasonality. HBoV showed a singular July epidemic at odds with the December convergence of the other viruses, together providing a preliminary basis for pathogen-tailored early warning and prevention in northern Chinese megacities. Co-infection occurred in 4.5% of positives, centered on HRV-containing combinations whose clinical severity remains to be defined. Set alongside pre-pandemic Chinese pediatric surveillance, these findings are broadly consistent with a post-pandemic pattern of intensified, temporally compressed winter transmission, while HBoV’s summer epidemic appears to be a locally distinctive feature warranting further study. If confirmed with higher-resolution, denominator-based data, these findings could inform a hospital-level operational calendar anchored on the December peak and the secondary July HBoV peak, climate-informed early warning tailored to each pathogen’s actual (seasonally adjusted) climate sensitivity, and a refined strategy for vaccination, RSV passive immunization and infection control. Such confirmation will require multi-center cohorts, denominator-based positivity metrics, weekly- or daily-resolution meteorology, and direct pre- versus post-pandemic comparison.

## Data Availability

The raw data supporting the conclusions of this article will be made available by the authors, without undue reservation.
